# Supplementary material for: Predicting visual function by interpreting a neuronal wiring diagram
Source: Nature. 2024 Oct 2;634(8032):113–23. doi: 10.1038/s41586-024-07953-5 (PMC11446822; doi:10.1038/s41586-024-07953-5)
Supplement: Supplementary file 6 — CRF and ERF predictions for individual TmY4 and TmY9 cells. Analogous to Supplementary Data 3, but for TmY target types. Shown are the top four monosynaptic pathways, the strongest pathway passing through each of the top ten intermediary types (ranking from Extended Data Fig. 7), and the trisynaptic pathway Tm1–TmY–Dm3–TmY (see the section entitled Prediction of spatial normalization). [file 41586_2024_7953_MOESM6_ESM.zip › DataS4/TmY9q/720575940638755910.pdf]

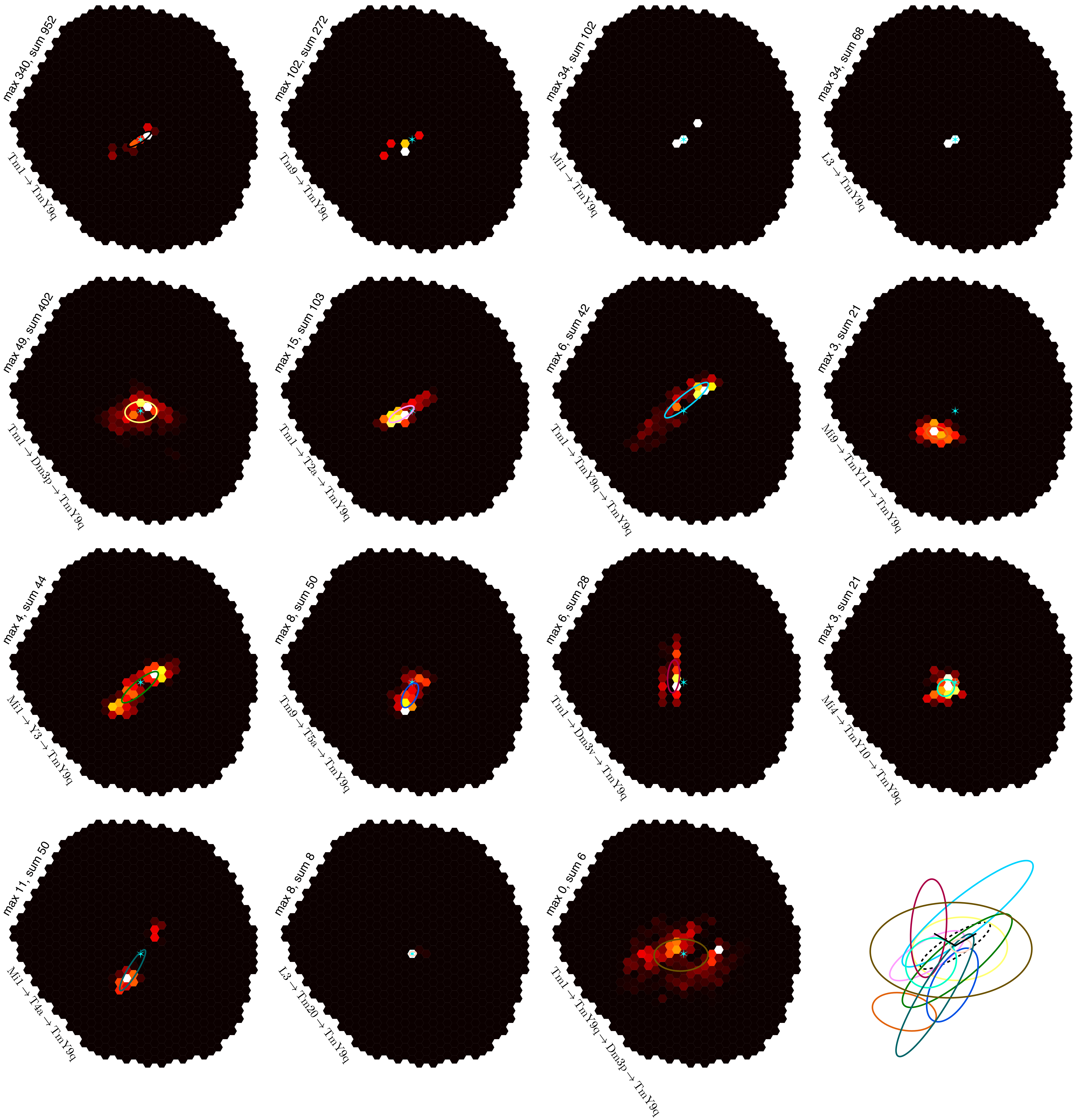
max 340, sum 952
Tm1 → TmY9q
max 102, sum 272
Tm9 → TmY9q
max 34, sum 102
Mi1 → TmY9q
max 34, sum 68
L3 → TmY9q
max 49, sum 402
Tm1 → Dm3p → TmY9q
max 15, sum 103
Tm1 → T2a → TmY9q
max 6, sum 42
Tm1 → TmY9q → TmY9q
max 3, sum 21
Mi9 → TmY11 → TmY9q
max 4, sum 44
Mi1 → Y3 → TmY9q
max 8, sum 50
Tm9 → T5a → TmY9q
max 6, sum 28
Tm1 → Dm3v → TmY9q
max 3, sum 21
Mi4 → TmY10 → TmY9q
max 11, sum 50
Mi1 → T4a → TmY9q
max 8, sum 8
L3 → Tm20 → TmY9q
max 0, sum 6
Tm1 → TmY9q → Dm3p → TmY9q
